# Prognostic Impact of Sarcopenia and Skeletal Muscle Loss During Neoadjuvant Chemoradiotherapy in Esophageal Cancer

**DOI:** 10.3390/cancers12040925

**Published:** 2020-04-10

**Authors:** Han Gyul Yoon, Dongryul Oh, Yong Chan Ahn, Jae Myoung Noh, Hongryull Pyo, Won Kyung Cho, Yun Mi Song, Minsu Park, Na Young Hwang, Jong-Mu Sun, Hong Kwan Kim, Jae Ill Zo, Young Mog Shim

**Affiliations:** 1 Department of Radiation Oncology, Samsung Medical Center, Sungkyunkwan University School of Medicine, Seoul 06351, Korea; hangyul.yoon@samsung.com (H.G.Y.); ycahn.ahn@samsung.com (Y.C.A.); jm2.noh@samsung.com (J.M.N.); hr.pyo@samsung.com (H.P.); wklove.cho@samsung.com (W.K.C.); 2 Department of Family Medicine, Samsung Medical Center, Sungkyunkwan University School of Medicine, Seoul 06351, Korea; yunmi.song@samsung.com; 3 Department of Statistics, Keimyung University, Daegu 42601, Korea; minsu.park51@kmu.ac.kr; 4 Statistics and Data Center, Samsung Medical Center, Sungkyunkwan University School of Medicine, Seoul 06351, Korea; ny.hwang@sbri.co.kr; 5 Department of Medicine, Division of Hematology-Oncology, Samsung Medical Center, Sungkyunkwan University School of Medicine, Seoul 06351, Korea; jongmu.sun@samsung.com; 6 Department of Thoracic and Cardiovascular Surgery, Samsung Medical Center, Sungkyunkwan University School of Medicine, Seoul 06351, Korea; hkts.kim@samsung.com (H.K.K.); jayl.zo@samsung.com (J.I.Z.); youngmog.shim@samsung.com (Y.M.S.)

**Keywords:** esophageal cancer, neoadjuvant chemoradiotherapy, sarcopenia, skeletal muscle loss, nutrition, inflammation

## Abstract

Backgrounds: The relationship between sarcopenia, characterized by loss of muscle mass and strength, and survival outcomes of esophageal cancer is controversial. This study aimed to assess the effect of sarcopenia and skeletal muscle loss on overall survival (OS) and recurrence-free survival (RFS) of esophageal cancer patients. Methods: We retrospectively collected the medical records of 248 male patients diagnosed with squamous cell esophageal cancer and who underwent neoadjuvant chemoradiotherapy (NACRT) followed by surgery. We measured the cross-sectional area of the skeletal muscle at the L3 vertebra level using computed tomography images and calculated the skeletal muscle index (SMI). Sarcopenia was defined as SMI <52.4 cm^2^/m^2^, and excessive muscle loss was defined as SMI change <−10.0%/50 days during NACRT. Moreover, laboratory test results, such as albumin, prognostic nutritional index (PNI), neutrophil-to-lymphocyte ratio (NLR), and platelet-to-lymphocyte ratio (PLR) before and after NACRT, were collected. Results: In the univariable Cox analysis, pre- (*p* = 0.689) and post-radiotherapy (RT) sarcopenia (*p* = 0.669) were not associated with OS. However, excessive muscle loss had a significant association with OS in both the univariable and multivariable analyses (all *p* = 0.001). Excessive muscle loss was also related to RFS in both the univariable (*p* = 0.011) and multivariable (*p* = 0.022) Cox analysis. Patients with excessive muscle loss had significantly lower levels of post-RT albumin (*p* < 0.001) and PNI (*p* < 0.001), higher levels of post-RT NLR (*p* = 0.031) and PLR (*p* = 0.071), larger decrease in albumin (*p* < 0.001) and PNI (*p* < 0.001) after NACRT, and larger increase in NLR (*p* = 0.051) and PLR (*p* = 0.088) after NACRT than in those with non-excessive muscle loss. Conclusion: Excessive muscle loss rather than pre- and post-RT sarcopenia was a significant prognostic factor for OS and RFS, and it was also related to nutritional and inflammatory markers.

## 1. Introduction 

Esophageal cancer is the sixth leading cause of cancer-related deaths worldwide [1]. Although the combination of neoadjuvant chemoradiotherapy (NACRT) and surgery improved overall survival (OS) and was adopted as a standard treatment for advanced thoracic esophageal cancer [2], esophageal cancer remains an aggressive and fatal disease [3]. Almost all patients treated with NACRT have malnutrition owing to poor oral intake and dysphagia during the treatment. Malnutrition is a critical issue that contributes to poor treatment outcomes in esophageal cancer patients [4]. 

Recent studies on relationships among nutrition, inflammation, and cancer have revealed that nutritional status affects the immunologic reaction and treatment response to cancer therapy [5]. Severe malnutrition and cachexia can entail chronic inflammation in cancer patients, which is related to poor prognosis [6]. As a result, clinicians and researchers have recently focused on sarcopenia, a condition characterized by the loss of muscle mass and strength in cancer patients [7]. Sarcopenia is associated with multiple medical issues such as disability, prolonged hospitalization, increased inflammatory marker levels, postoperative complications, falls, and poor quality of life [8,9,10,11]. In addition, using computed tomography (CT)-based analysis, which is one of the most commonly used methods for evaluating muscle mass, recent studies reported that sarcopenia is a risk factor associated with poor survival of patients with various types of tumors [12,13,14]. 

In line with these trends, the effect of sarcopenia on the prognosis of esophageal cancer has been widely studied [15,16,17,18,19,20]. However, the association between sarcopenia and patient survival remains controversial. While several studies showed that sarcopenia is a risk factor for poor survival [20,21,22], some others did not [17,18,23]. Furthermore, because most patients included in these studies received chemotherapy rather than chemoradiotherapy as a neoadjuvant treatment (NAT) [18,20,21,22], changes in skeletal muscle mass and laboratory test results before and after NACRT have not been well-studied. Therefore, this study aimed to assess whether sarcopenia and loss of skeletal muscle affected survival outcomes of esophageal cancer patients who received NACRT followed by surgery. In addition, we analyzed clinicopathologic features and laboratory test values associated with nutrition and inflammation in patients with poor prognosis and investigated their relationship with sarcopenia and loss of skeletal muscle.

## 2. Materials and Methods

### 2.1. Study Design and Data Collection

We retrospectively reviewed the medical records of esophageal cancer patients who underwent NACRT followed by surgery between 2005 and 2017 at the Samsung Medical Center, Seoul, Republic of Korea. This study was approved by our Institutional Review Board (IRB #2019-02-070-002) and was performed in accordance with the guidelines of the Declaration of Helsinki. The inclusion criteria were (i) completion of NACRT and (ii) having 18F-fluorodeoxyglucose positron emission tomography-computed tomography (PET-CT) data for both the pre- and post-radiotherapy (RT) period. Of 311 patients initially enrolled, 48 were excluded either for having received palliative surgery (*n* = 3) or for the impossibility of the in-house software to calculate skeletal muscle mass (*n* = 45). In addition, female patients (*n* = 15) were excluded because they are physiologically different from men, have relatively low baseline skeletal muscle mass, and were a group with a small sample size that would necessarily require statistical adjustment. The final statistical analysis was performed with 248 patients. 

We obtained clinicopathologic features and pre- and post-RT laboratory test results of the included patients. Pre-RT blood test values were measured on the day nearest to the start of RT, while post-RT blood test values were measured on the day nearest to the end of RT. Laboratory test results included absolute counts of white blood cells (/μL), absolute neutrophil count (ANC, /μL), absolute lymphocyte count (ALC, /μL), platelet count (/μL), and albumin (g/dL) levels. The neutrophil-to-lymphocyte ratio (NLR) and platelet-to-lymphocyte ratio (PLR) were calculated as ANC/ALC and platelet count/ALC, respectively. In addition, we calculated the prognostic nutritional index (PNI), which is associated with the survival outcome and nutritional status of cancer patients, including esophageal cancer patients [24,25]. PNI was calculated as (10 × albumin [g/dL] + 0.005 × ALC) [26]. 

### 2.2. Treatment Scheme

The patients received 5 weeks of RT with a total dose ranging from 40 to 50 Gy (1.8–2.15 Gy per fraction), and most of them received (89.9%) 44 Gy in 22 fractions (89.9%). Gross tumor volume (GTV) included primary tumor and metastatic lymph nodes (LNs) based on endoscopic observations, CT, and PET-CT. The clinical target volume (CTV) of the primary tumor was defined as primary GTV plus a 0.5-cm margin in the circumferential direction and 2–3-cm margin in the craniocaudal direction. The nodal CTV was defined by adding a 1-cm margin in all directions from the metastatic LNs, and elective nodal regions were not included. Considering possible errors owing to daily setup variations and respiratory motion, the planning target volume was defined as CTV plus a 0.5–0.7 cm margin in all directions. Three or four beam arrangements were typically used to cover the target volumes, and RT was administered using 4–10 MV photon beams from linear accelerators. Three-dimensional conformal RT and intensity-modulated RT were administered to 198 (79.8%) and 50 patients (20.2%), respectively.

Along with RT, two cycles of concurrent chemotherapy were delivered intravenously to the patients 3 weeks apart. The first cycle was planned to start on the initial day of RT, and the regimen of each cycle was composed of 5-fluorouracil 1000 mg/m^2^/day for 4 consecutive days and cisplatin 60 mg/m^2^/day on the first day. After completing NACRT, all patients underwent transthoracic esophagectomy with two- or three-field lymphadenectomy. Two-field (thoracic and abdominal) and three-field (cervical, thoracic, and abdominal) LN dissections were performed in lower thoracic esophageal cancer and upper or mid-thoracic esophageal cancer patients, respectively.

### 2.3. Skeletal Muscle Mass Assessment and Definition of Sarcopenia

To evaluate the body composition of patients, we used CT images of two consecutive PET-CT performed before and after NACRT (all post-RT PET-CT was performed before surgery). We measured the cross-sectional area (cm^2^) of the skeletal muscle in CT images taken at the level of the third lumbar (L3) vertebra using the in-house software based on MATLAB version R2014a (Mathworks Inc., Natick, MA, USA). The software used in this study is an open-source tool, which is available at the following URL (https://sourceforge.net/projects/muscle-fatarea-measurement/). The skeletal muscle area obtained was then divided by the square of the height (m^2^), and this value was defined as the skeletal muscle index (SMI). The cut-off value of SMI for defining sarcopenia was 52.4 cm^2^/m^2^ according to the results of a previous population-based study [27]. After the calculation of pre-RT SMI and post-RT SMI, the SMI change before and after NACRT (ΔSMI) was expressed as a percentage relative to pre-RT SMI (ΔSMI [%]). Because there was variation among patients in the time interval between pre- and post-RT PET-CT scans (median 70 days; interquartile range [IQR] 63–70 days), ΔSMI (%) was divided by the number of interval days (days) multiplied by 50 (ΔSMI [%]/50 days). 

### 2.4. Statistical Analysis 

The primary endpoint of this study was OS. The duration of OS was calculated from the initial date of NACRT to the date of the last follow-up or death. The secondary endpoint was recurrence-free survival (RFS). The duration of RFS was calculated from the initial date of NACRT to the date of the last follow-up or recurrence or death. The survival rates were calculated using the Kaplan–Meier method and were compared by log-rank tests. A Cox proportional hazards regression model was used for both univariable and multivariable analyses. Factors with a *p*-value < 0.05 on univariable analysis or factors considered clinically relevant were used in the multivariable analysis; in addition, the presence of pre-RT sarcopenia was included in the multivariable analysis to compensate for the effect of pre-RT SMI on ΔSMI (%)/50 days. 

To determine the optimal cut-off value of ΔSMI (%)/50 days for the segregation of patients into good or poor prognosis groups for OS, Maxstat, a maximal Chi-square method in R version 3.5.3 (R Development Core Team, Vienna, Austria, http://www.r-project.org) was used. The calculated cut-off value was rounded off to the first decimal place, and the log-rank test was used to measure the strength of the groupings. According to this method, −10.0 (%/50 days) was determined as the optimal cut-off value (*p* < 0.001). Therefore, we classified patients in the “excessive muscle loss group (ΔSMI (%)/50 days <−10.0%/50 days)” or the “non-excessive muscle loss group (ΔSMI (%)/50 days ≥−10.0%/50 days).”

After classifying the patients into two groups, the Chi-square test or Fisher’s exact test was used to compare the categorical variables of each group. All continuous variables were described as mean ± standard deviation, and their normality was examined using the Shapiro–Wilk test. After the normality test, Student’s *t*-test was used to compare the normally distributed continuous variables and the Wilcoxon rank-sum test was used to compare non-normally distributed variables. All statistical analyses in this study were performed using the SAS software version 9.4 (Cary, NC, USA), SPSS software version 24.0 (IBM Corporation, Armonk, NY, USA), and R version 3.5.3 (R Development Core Team, Vienna, Austria, http://www.r-project.org).

## 3. Results

### 3.1. Patient Characteristics 

The clinicopathologic characteristics and baseline laboratory test results for all patients are shown in Table 1. All patients were diagnosed with squamous cell carcinoma, and the mean age was 63.46 ± 7.63 years. The majority of patients (94.0%) had an Eastern Cooperative Oncology Group (ECOG) performance status of 1. The tumor location was upper, middle, and lower esophagus in 66 (26.6%), 117 (47.2%), and 65 (26.2%) patients, respectively. The commonest clinical T stages were cT3 (73.0%) and cT2 (19.4%), and the commonest clinical N stages were cN1 (55.6%) and cN2 (36.3%). R0 resections were achieved in 236 (95.2%) patients, and 70 (28.2%) patients experienced pathologic complete response. Regarding sarcopenia, 156 (62.9%) patients had sarcopenia before NACRT and 207 (83.5%) had sarcopenia after NACRT. The mean ΔSMI (%)/50 days was −6.47 ± 6.11%/50 days, and 70 (28.2%) patients were classified in the excessive muscle loss group. 

### 3.2. Survival Analysis 

**Figure 1:**
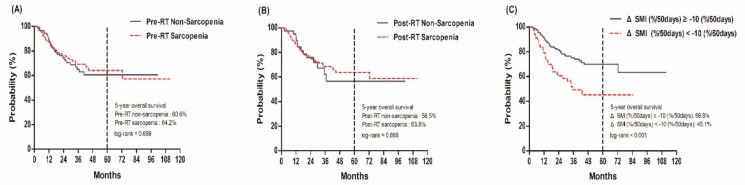
Overall survival between (**A**) Pre-RT non-sarcopenia vs. Pre-RT sarcopenia (**B**) Post-RT non-sarcopenia vs. Post-RT sarcopenia (**C**) ΔSMI (%/50days) ≥−10.0 (%/50days) vs. <−10.0 (%/50days).

During the median follow-up of 26.3 months (range, 2.4–111.3 months), 74 (29.8%) patients died, and the 5-year OS rate was 62.5%. On initial survival analysis using the Kaplan–Meier estimate, there was no significant difference in the 5-year OS rate between the pre-RT sarcopenia and pre-RT non-sarcopenia groups (64.2% vs. 60.6%, *p* = 0.689) and between the post-RT sarcopenia and post-RT non-sarcopenia groups (63.8% vs. 56.5%, *p* = 0.668). In contrast, the excessive muscle loss group had a significantly lower 5-year OS rate than the non-excessive muscle loss group (45.1% vs. 69.8%, *p* < 0.001). The above results are shown in Figure 1.

The results of univariable and multivariable Cox analyses for OS are summarized in Table 2. In the univariable Cox analysis, pre-RT sarcopenia (hazard ratio (HR) = 0.910, 95% confidence interval (CI) 0.572–1.447, *p* = 0.689) and post-RT sarcopenia (HR = 0.877, 95% CI 0.482–1.598, *p* = 0.669) were not associated with OS, which shows the same tendency as the results in Figure 1. Conversely, ΔSMI (%)/50 days <−10.0%/50 days was significantly related to OS (HR = 2.242, 95% CI 1.415–3.554, *p* = 0.001). Moreover, cT3-4 stage (HR = 2.028, 95% CI 1.092–3.767, *p* = 0.025), cN2-3 stage (HR = 1.804, 95% CI 1.141–2.853, *p* = 0.012), ypT1-4 stage (HR = 3.276, 95% CI 1.952–5.498, *p* < 0.001), ypN+ stage (HR = 2.617, 95% CI 1.588–4.313, *p* < 0.001), R1-2 resection (HR = 8.021, 95% CI 4.140–15.538, *p* < 0.001), and baseline albumin (HR = 0.481, 95% CI 0.286–0.810, *p* = 0.006) were associated with OS. In the multivariable analysis, ΔSMI (%/50 days) <−10.0%/50 days remained a significant prognostic factor for OS (HR = 2.299, 95% CI 1.415–3.733, *p* = 0.001). Additionally, cN2-3 stage (HR = 1.671, 95% CI 1.029–2.712, *p* = 0.038), ypT1-4 stage (HR = 2.656, 95% CI 1.500–4.703, *p* = 0.001), ypN+ stage (HR = 1.994, 95% CI 1.175–3.384, *p* = 0.011), R1-2 resection (HR = 6.457, 95% CI 3.165–13.373, *p* < 0.001), and albumin (HR = 0.357, 95% CI 0.189–0.673, *p* = 0.002) were statistically significant factors for OS. 

The 5-year RFS of total patients was 47.4%. There was no significant difference in the RFS between the pre-RT sarcopenia and pre-RT non-sarcopenia groups (*p* = 0.875, 5-year RFS rate 48.3% vs. 45.8%) and between the post-RT sarcopenia and post-RT non-sarcopenia groups (*p* = 0.646, 5-year RFS rate 48.3% vs. 41.2%). In contrast, patients with the excessive muscle loss showed worse RFS than patients without the excessive muscle loss (*p* = 0.010, 5-year RFS rate 33.5% vs. 52.9%). The excessive muscle loss was also significantly related to RFS both in the univariable (HR = 1.622, 95% CI 1.119–2.350, *p* = 0.011) and multivariable (HR = 1.571, 95% CI 1.066–2.314, *p* = 0.022) Cox analyses, which are summarized in Supplementary Table S1. 

### 3.3. Comparison Between the Excessive and Non-Excessive Muscle Loss Groups

As the ΔSMI (%/50 days) <−10.0%/50 days was significantly associated with OS, clinicopathologic characteristics and laboratory test results were compared between the excessive and non-excessive muscle loss groups, which are summarized in Table 3 and Table 4. There was no significant difference in clinicopathologic characteristics, such as age, ECOG performance status, current smoking, location of the tumor, cT/N stage, ypT/N stage, and resection margin status, between the two groups. Moreover, pre-RT body mass index and SMI were not lower in the excessive muscle loss group than in the non-excessive muscle loss group. Pre-RT SMI was significantly higher in the excessive muscle loss group than in the non-excessive muscle loss group (51.70 ± 7.91 vs. 48.94 ± 7.81, *p* = 0.015).

On the contrary, significant differences were observed in the laboratory test results, especially with respect to post-RT values and in the differences in values before and after NACRT. Although the baseline pre-RT blood test results did not significantly differ between the two groups, patients with excessive muscle loss had significantly lower post-RT nutritional marker levels such as albumin (3.55 ± 0.44 vs. 3.87 ± 0.42, *p* < 0.001) and PNI (41.06 ± 6.58 vs. 44.51 ± 5.40, *p* < 0.001) than those with non-excessive muscle loss. Furthermore, the excessive muscle loss group showed a larger decrease in albumin (−0.73 ± 0.45 vs. −0.42 ± 0.45, *p* < 0.001) and PNI (−12.70 ± 7.49 vs. −8.84 ± 6.40, *p* < 0.001) after NACRT than the non-excessive muscle loss group. In addition, the excessive muscle loss group showed higher post-RT NLR (6.07 ± 9.76 vs. 3.48 ± 4.28, *p* = 0.031) and PLR (382.12 ± 508.48 vs. 239.72 ± 248.24, *p* = 0.071) along with a greater increase of NLR (3.36 ± 9.92 vs. 1.05 ± 4.39, *p* = 0.051) and PLR (255.00 ± 514.00 vs. 114.00 ± 244.00, *p* = 0.088) after NACRT than the non-excessive muscle loss group, although these tendencies were not statistically significant except for the post-RT NLR.

## 4. Discussion 

This study investigated the effect of sarcopenia and skeletal muscle loss during NACRT on survival outcomes of esophageal cancer patients who received NACRT followed by surgery. Pre- and post-RT sarcopenia were not associated with OS or RFS, but ΔSMI (%)/50 days <−10.0%/50 days had a significant association with poor OS and RFS in both univariable and multivariable analyses. Moreover, compared with non-excessive muscle loss, excessive muscle loss was associated with lower post-RT nutritional marker levels (albumin, PNI), higher post-RT inflammatory marker levels (NLR, PLR), larger decrease in nutritional marker levels after NACRT, and larger increase in inflammatory marker levels after NACRT.

Several studies have investigated the effect of sarcopenia on the prognosis of esophageal cancer. However, the results were contradictory [17,18,20,21,22,23]. In addition, only a few studies included patients who received NACRT instead of chemotherapy as a NAT, and none of these studies demonstrated a significant relationship between sarcopenia and OS [17,23]. Meanwhile, studies on the relationship between the amount of skeletal muscle loss and survival outcomes of esophageal cancer showed relatively consistent results for OS, although they still showed contradictory results for RFS [28,29,30,31,32]. Reisinger et al. showed that the amount of muscle mass loss during NACRT was associated with postoperative mortality in patients with stage III–IV tumors [28]. Kamitani et al. also found that ΔSMI (%) <−12.5% was a significant prognostic factor for OS, and pre- and post-NAT sarcopenia were not associated with OS [29]. Similar to these results, Järvinen et al. reported that a ΔSMI (%) <−2.98% during NAT was related to poor OS in the multivariable analysis, whereas post-NAT sarcopenia did not significantly affect OS and complication rates [30]. RFS was also analyzed in this study, but ΔSMI (%) <−2.98%, pre- and post-NAT sarcopenia all did not affect RFS. Mayanagi et al. also investigated the prognostic significance of skeletal muscle wasting during NAT for RFS and OS. Skeletal muscle wasting was an independent prognostic factor for both RFS and OS in multivariable analysis [31]. 

This study demonstrated the correlation of inflammatory/nutritional markers with skeletal muscle changes during NACRT in esophageal cancer patients. There are some similarities with prior studies, which identified the association between systemic inflammation and skeletal muscle mass in various types of tumors. In a retrospective study with advanced pancreatic cancer patients, ΔSMI (%) <−10.0% at the first evaluation was associated with a larger increase of NLR and poorer OS than ΔSMI (%) ≥−10.0% [33]. Another prospective study of 670 gastric cancer patients showed that NLR and PLR were significantly higher in the preoperative sarcopenia group than in the non-sarcopenia group [34]. In addition, Kim et al. reported a significant correlation between SMI and lymphocyte count, albumin, NLR, and C-reactive protein (CRP) in small cell lung cancer patients. Similarly, in esophageal cancer, Matsunaga et al. reported that patients with preoperative sarcopenia tended to have relatively higher NLR, CRP-to-albumin ratio, and modified Glasgow prognostic score than those without preoperative sarcopenia, although the effect of sarcopenia on NLR was not statistically significant (*p* = 0.052). To the best of our knowledge, no other studies have investigated the relationship between laboratory test results and SMI in esophageal cancer patients. 

Until now, little was known about the interactions among skeletal muscle loss, nutrition, and inflammation in cancer patients. The current study showed that skeletal muscle loss is associated with decreased nutritional marker level and increased inflammatory marker level. Additionally, skeletal muscle loss is correlated with OS and RFS. However, it is difficult to conclude the cause and effect relation between them. The hypothesis of the relationships might be deduced from the results of this and other studies. One of the key factors is myokine, which is a cytokine produced and released by skeletal muscles [35]. It was recently discovered that myokines mediate anti-inflammatory response and have potential anti-cancer functions [36]. Myokines, such as interleukin (IL)-6, can have anti-tumorigenic effects by interacting with NK cells and inducing the production of IL-1 receptor antagonist and IL-10 by blood mononuclear cells, which are molecules with anti-inflammatory effects [37,38]. In other words, skeletal muscle status affects tumorigenesis and systemic inflammation. Conversely, tumor-derived factors and pro-inflammatory cytokines can inhibit protein synthesis and muscle regeneration and trigger anorexia, protein degradation, and apoptosis of myofibers [39]. Similarly, several inflammation-induced molecular pathways can promote involuntary muscle loss, which is mediated by factors such as tumor necrosis factor (TNF)-α, NF-κB, and calcium-dependent enzymes [40,41]. Moreover, inflammation can contribute to tumorigenesis and tumor progression via multiple mechanisms [42,43]. In summary, these results suggest that muscle degradation may be associated with inflammatory reactions, and impairment of anti-tumor immunity, although further results from prospective studies and basic researches are in need to prove this association. 

However, preventing skeletal muscle loss is quite challenging in esophageal cancer. Approximately 80% of esophageal cancer patients have malnutrition before the start of treatment, mainly because of dysphagia and anorexia [44,45]. Considering that malnutrition is a critical risk factor for skeletal muscle loss, these problematics become more significant [46]. Moreover, despite its survival benefit, NACRT itself can deteriorate the nutrition and muscle status of esophageal cancer patients. Cytotoxic agents such as cisplatin can promote muscle wasting by disrupting molecular signaling pathways [47,48], and NACRT can cause side effects such as nausea, vomiting, anorexia, dysphagia, and esophagitis [49,50]. In addition, NAT reduces physical fitness and muscle strength in esophageal cancer patients [51,52]. Therefore, adequate expert support for nutrition and physical activity during NAT is needed to improve the prognosis and quality of life in these patients [53,54,55]. 

This study has some limitations. First, we retrospectively collected and reviewed data from a single institution. Second, owing to the retrospective study design, several laboratory profiles such as CRP, erythrocyte sedimentation rate, TNF-α, IL-1, and IL-6 could not be investigated. Further sophisticated analyses could have been performed if data for these parameters were available. Third, all included patients were men who were diagnosed with squamous cell carcinoma. Although the majority of esophageal cancer patients in Korea are men with squamous cell carcinoma [56], further studies that include women and various histologic types of esophageal cancer are needed. Finally, because there is no definite consensus on the cut-off value of SMI for defining sarcopenia, this study adopted the value proposed by a previous population-based study [27], which was used in several recent studies on cancer patients [57,58,59]. However, future studies are needed to determine the exact cut-off value of SMI for defining sarcopenia considering the characteristics of East Asian individuals.

## 5. Conclusions

In conclusion, excessive muscle loss rather than pre- and post-RT sarcopenia was a significant factor for OS and RFS in this study. Moreover, excessive muscle loss was related to changes in nutritional and inflammatory marker levels, which may imply a possible linkage between nutrition, inflammation, and tumor progression. Based on the study results, this study suggests that interventions to prevent excessive muscle loss, such as nutritional support and regular exercise, may improve therapeutic outcomes in esophageal cancer patients. Further studies are needed to validate our study results and address the beneficial effects of appropriate expert intervention for nutrition, physical activity, and muscle mass maintenance during the treatment of this type of cancer.

## Figures and Tables

**Table 1:** Patient characteristics, with baseline laboratory test results (*n* = 248).

Cliniocopathologic Characteristics ^a^	
**Age (years)**	63.46 ± 7.63
**ECOG performance status**	
0 1 2	11 (4.4%) 233 (94.0%) 4 (1.6%)
**Smoking**	
Current smoker Ex-smoker Non-smoker	138 (55.6%) 92 (37.1%) 18 (7.3%)
**Location**	
Upper Middle Lower	66 (26.6%) 117 (47.2%) 65 (26.2%)
**cT stage**	
cT1 cT2 cT3 cT4	13 (5.2%) 48 (19.4%) 181 (73.0%) 6 (2.4%)
**cN stage**	
cN0 cN1 cN2 cN3	14 (5.6%) 138 (55.6%) 90 (36.3%) 6 (2.4%)
**ypT stage**	
ypT0/Tis ypT1 ypT2 ypT3 ypT4	117 (47.2%) 31 (12.5%) 44 (17.7%) 50 (20.2%) 6 (2.4%)
**ypN stage**	
ypN0 ypN1 ypN2 ypN3	121 (48.8%) 81 (32.7%) 29 (11.7%) 17 (6.9%)
**ypCR**	
Yes No	70 (28.2%) 178 (71.8%)
**Resection Margin**	
R0 R1–R2	236 (95.2%) 12 (4.8%)
**BMI (kg/m^2^)**	22.92 ± 2.85
**SMI**	
Pre-RT Post-RT ΔSMI (%/50days)	49.72 ± 7.92 45.10 ± 7.57 −6.47 ± 6.11
**Pre-RT sarcopenia**	
Yes No	156 (62.9%) 92 (37.1%)
**Post-RT sarcopenia**	
Yes No	207 (83.5%) 41 (16.5%)
**Baseline laboratory test results^a^**	
WBC (*10^3^/µL)	7.79 ± 2.35
ANC (*10^3^/µL)	4.84 ± 2.09
ALC (*10^3^/µL)	2.11 ± 0.67
Platelet (*10^3^/µL)	245.41 ± 70.08
**Albumin (g/dl)**	4.29 ± 0.36
**NLR**	2.50 ± 1.42
**PLR**	125.83 ± 52.77
**PNI**	53.45 ± 5.48

ECOG, Eastern Cooperative Oncology Group; CR, complete response; RT, radiation therapy; BMI, Body Mass Index; SMI, Skeletal Muscle Index; WBC, white blood cell count; ANC, absolute neutrophil count; ALC, absolute lymphocyte count; NLR, neutrophil to lymphocyte ratio; PLR, platelet to lymphocyte ratio; PNI, Prognostic Nutritional Index, calculated as (10 × albumin [g/dL] + 0.005 × ALC). ^a^ Continuous variables are described as mean ± standard deviation, while nominal variables are described as number (%).

**Table 2:** Univariable and multivariable analysis for overall survival.

Variables	Univariable Analysis		Multivariable Analysis	
	HR (95% CI)	*p*-Value	HR (95% CI)	*p*-Value
**Age (years)**	0.982 (0.953–1.013)	0.258	0.996 (0.961–1.031)	0.814
**ECOG performance status**				
0–1 2	1 1.239 (0.172–8.928)	- 0.832		
**Current smoking**				
No Yes	1 1.145 (0.721–1.819)	- 0.565	1 1.577 (0.918–2.708)	- 0.099
**Location**				
Upper Middle Lower	1 0.907 (0.539–1.525) 0.595 (0.313–1.128)	- 0.712 0.112		
**cT stage**				
cT1-2 cT3-4	1 2.028 (1.092–3.767)	- 0.025	1 1.109 (0.562–2.188)	- 0.767
**cN stage**				
cN0-1 cN2-3	1 1.804 (1.141–2.853)	- 0.012	1 1.671 (1.029–2.712)	- 0.038
**ypT stage**				
ypT0/Tis ypT1-4	1 3.276 (1.952–5.498)	- <0.001	1 2.656 (1.500–4.703)	- 0.001
**ypN stage**				
ypN0 ypN+	1 2.617 (1.588–4.313)	- <0.001	1 1.994 (1.175–3.384)	- 0.011
**Resection margin**				
R0 R1-2	1 8.021 (4.140–15.538)	- <0.001	1 6.457 (3.165–13.173)	- <0.001
**BMI (kg/m^2^)**	0.948 (0.872–1.030)	0.205		
**Pre-RT sarcopenia**				
No Yes	1 0.910 (0.572–1.447)	- 0.689	1 1.009 (0.619–1.644)	- 0.973
**Post-RT sarcopenia**				
No Yes	1 0.877 (0.482–1.598)	- 0.669		
**ΔSMI (%/50days)**				
≥ −10 (%/50days) < −10 (%/50days)	1 2.242 (1.415–3.554)	- 0.001	1 2.299 (1.415–3.733)	- 0.001
**Albumin (g/dL)**	0.481 (0.286–0.810)	0.006	0.357 (0.189–0.673)	0.002
**NLR**	0.972 (0.813–1.162)	0.755		
**PLR**	1.002 (0.998–1.006)	0.298		
**PNI**	0.972 (0.932–1.014)	0.188		

HR, hazard ratio; CI, confidence interval; ECOG, Eastern Cooperative Oncology Group; RT, radiation therapy; BMI, Body Mass Index; SMI, Skeletal Muscle Index; NLR, neutrophil to lymphocyte ratio; PLR, platelet to lymphocyte ratio; PNI, Prognostic Nutritional Index.

**Table 3:** Comparison of clinicopathologic characteristics between excessive muscle loss group and non-excessive muscle loss group.

Variables	Non-Excessive Muscle Loss Group (*n* = 178)	Excessive Muscle Loss Group ^a^ (*n* = 70)	*p*-Value
**Age (years)**	62.49 ± 7.58	64.26 ± 8.14	0.108 ^b^
**ECOG performance status**			0.579 ^c^
0–1 2	174 (97.8%) 4 (2.3%)	70 (100.0%) 0 (0.0%)	
**Current smoking**			0.206
No Yes	74 (41.6%) 104 (58.4%)	36 (51.4%) 34 (48.6%)	
**Location**			0.308
Upper Middle Lower	48 (27.0%) 88 (49.4%) 42 (23.6%)	18 (25.7%) 29 (41.4%) 23 (32.9%)	
**cT stage**			0.223
cT1-2 cT3-4	48 (27.0%) 130 (73.0%)	13 (18.6%) 57 (81.4%)	
**cN stage**			0.684
cN0-1 cN2-3	111 (62.4%) 67 (37.6%)	41 (58.6%) 29 (41.4%)	
**ypT stage**			0.484
ypT0/Tis ypT1-4	81 (45.5%) 97 (54.5%)	36 (51.4%) 34 (48.6%)	
**ypN stage**			0.302
ypN0 ypN+	91 (51.1%) 87 (48.9%)	30 (42.9%) 40 (57.1%)	
**ypCR**			0.479
No Yes	125 (70.2%) 53 (29.8%)	53 (75.7%) 17 (24.3%)	
**Resection margin**			0.526 ^c^
R0 R1-2	170 (95.5%) 8 (4.5%)	65 (92.9%) 5 (7.1%)	

ECOG, Eastern Cooperative Oncology Group; CR, Complete response. ^a^ Excessive muscle group was defined as ΔSMI (%/50days) <−10 (%/50days). ^b^ Calculated by Student’s *t*-test. ^c^ Calculated by Fisher’s exact test.

**Table 4:** Comparison of body components and laboratory test results between excessive muscle loss group and non-excessive muscle loss group.

Variables	Total Patients ^a^ (*n* = 248)	Non-Excessive Muscle Loss Group ^a^ (*N* = 178)	Excessive Muscle Loss Group ^a^ (*n* = 70)	*p*-Value
**BMI**				
Pre-RT Post-RT ΔRT	22.92 ± 2.85 22.15 ± 2.87 −0.78 ± 1.47	22.71 ± 2.80 22.36 ± 2.84 −0.35 ± 1.24	23.47 ± 2.94 21.61 ± 2.89 −1.86 ± 1.49	0.067 ^b^ 0.068 ^b^ <0.001 ^b^
**SMI**				
Pre-RT Post-RT ΔRT	49.72 ± 7.92 45.10 ± 7.57 −4.62 ± 4.36	48.94 ± 7.81 46.24 ± 7.39 −2.70 ± 3.18	51.70 ± 7.91 42.20 ± 7.31 −9.49 ± 2.91	0.015 ^b^ <0.001 <0.001
WBC (*10^3^/µL)				
Pre-RT Post-RT ΔRT	7.79 ± 2.35 4.73 ± 2.39 −3.05 ± 3.26	7.63 ± 2.32 4.69 ± 2.42 −2.94 ± 3.23	8.19 ± 2.38 4.84 ± 2.32 −3.35 ± 3.33	0.060 0.384 0.410
ANC (*10^3^/µL)				
Pre-RT Post-RT ΔRT	4.84 ± 2.09 2.92 ± 2.17 −1.92 ± 2.91	4.71 ± 2.02 2.84 ± 2.19 −1.87 ± 2.86	5.18 ± 2.22 3.14 ± 2.13 −2.05 ± 3.05	0.063 0.137 0.597
ALC (*10^3^/µL)				
Pre-RT Post-RT ΔRT	2.11 ± 0.67	2.09 ± 0.64	2.17 ± 0.75	0.295
	1.14 ± 0.65 −0.97 ± 0.87	1.16 ± 0.61 −0.93 ± 0.78	1.10 ± 0.74 −1.07 ± 1.06	0.274 0.116
Platelet (*10^3^/µL)				
Pre-RT Post-RT ΔRT	245.41 ± 70.08	241.81 ± 69.55	246.82 ± 70.43	0.571
	198.25 ± 62.96 −47.16 ± 63.18	194.70 ± 62.03 −52.10 ± 59.70	207.26 ± 64.86 −34.60 ± 70.20	0.068 0.083
**Albumin (g/dL)**				
Pre-RT Post-RT ΔRT	4.29 ± 0.36	4.29 ± 0.36	4.29 ± 0.39	0.991
	3.78 ± 0.45 −0.51 ± 0.47	3.87 ± 0.42 −0.42 ± 0.45	3.55 ± 0.44 −0.73 ± 0.45	<0.001 <0.001
**NLR**				
Pre-RT Post-RT ΔRT	2.50 ± 1.42	2.42 ± 1.23	2.71 ± 1.81	0.272
	4.21 ± 6.41 1.70 ± 6.51	3.48 ± 4.28 1.05 ± 4.39	6.07 ± 9.76 3.36 ± 9.92	0.031 0.051
**PLR**				
Pre-RT Post-RT ΔRT	125.83 ± 52.77	125.46 ± 43.40	126.78 ± 71.68	0.188
	279.91 ± 347.15 154.08 ± 347.26	239.72 ± 248.24 114.00 ± 244.00	382.12 ± 508.48 255.00 ± 514.00	0.071 0.088
**PNI**				
Pre-RT Post-RT ΔRT	53.45 ± 5.48	53.35 ± 5.37	53.73 ± 5.79	0.606
	43.53 ± 5.95 −9.92 ± 6.93	44.51 ± 5.40 −8.84 ± 6.40	41.06 ± 6.58 −12.70 ± 7.49	<0.001 <0.001

RT, Radiation Therapy; WBC, White Blood Cell count; ANC, Absolute Neutrophil Count; ALC, Absolute Lymphocyte Count; NLR, Neutrophil to Lymphocyte Ratio; PLR, Platelet to Lymphocyte Ratio; PNI, Prognostic Nutritional Index; SMI, Skeletal Muscle Index. ^a^ All variables are described as mean ± standard deviation, and ΔRT was calculated as (Post-RT value – Pre-RT value). ^b^ Calculated by Student’s t-test.

## Supplementary Materials

The following are available online at https://www.mdpi.com/2072-6694/12/4/925/s1, Table S1. Univariable and multivariable analysis for recurrence-free survival.
