# Supplementary material for: Prognostic Impact of Sarcopenia and Skeletal Muscle Loss During Neoadjuvant Chemoradiotherapy in Esophageal Cancer
Source: Cancers (Basel). 2020 Apr 10;12(4):925. doi: 10.3390/cancers12040925 (PMC7226603; doi:10.3390/cancers12040925)
Supplement: Supplementary file 1 [file cancers-12-00925-s001.pdf]

Supplementary Materials

Table S1. Univariable and multivariable analysis for recurrence-free survival.

Variables	Univariable Analysis		Multivariable Analysis	
	HR (95% CI)	*p*-value	HR (95% CI)	*p*-value
**Age (years)**	0.961 (0.936–0.967)	0.003	0.985 (0.959–1.012)	0.270
**ECOG performance status**				
0–1	1	-		
2	2.216 (0.704–6.978)	0.174		
**Current smoking**				
No	1	-	1	-
Yes	0.990 (0.694–1.413)	0.957	1.141 (0.755–1.724)	0.531
**Location**				
Upper	1	-		
Middle	1.274 (0.777–2.089)	0.336		
Lower	1.324 (0.844–2.079)	0.222		
**cT stage**				
cT1-2	1	-	1	-
cT3-4	1.731 (1.091–2.747)	0.020	1.126 (0.680–1.863)	0.645
**cN stage**				
cN0-1	1	-	1	-
cN2-3	2.072 (1.452–2.957)	<0.001	1.816 (1.248-2.641)	0.002
**ypT stage**				
ypT0/Tis	1	-	1	-
ypT1-4	2.716 (1.848–3.992)	<0.001	2.200 (1.437–3.368)	<0.001
**ypN stage**				
ypN0	1	-	1	-
ypN+	2.573 (1.758–3.766)	<0.001	2.057 (1.368–3.094)	0.001
**Resection margin**				
R0	1	-	1	-
R1-2	5.725 (3.180–10.306)	<0.001	3.259 (1.749–6.074)	<0.001
**BMI (kg/m²)**				
**Pre-RT sarcopenia**				
No	1	-	1	-
Yes	1.030 (0.716–1.481)	0.875	1.070 (0.732–1.563)	0.728
**Post-RT sarcopenia**				
No	1	-		
Yes	1.116 (0.698–1.786)	0.647		
**ΔSMI (%/50days)**				
≥ −10 (%/50days)	1	-	1	-
< −10 (%/50days)	1.622 (1.119–2.350)	0.011	1.571 (1.066–2.314)	0.022
**Albumin (g/dL)**	0.570 (0.371–0.878)	0.011	0.457 (0.275–0.761)	0.003
**NLR**	0.963 (0.842–1.101)	0.580		
**PLR**	1.002 (0.999–1.005)	0.124		
**PNI**	0.976 (0.944–1.008)	0.142		

HR, Hazard Ratio; CI, Confidence Interval; ECOG, Eastern Cooperative Oncology Group; RT, Radiation Therapy; BMI, Body Mass Index; SMI, Skeletal Muscle Index; NLR, Neutrophil to Lymphocyte Ratio; PLR, Platelet to Lymphocyte Ratio; PNI, Prognostic Nutritional Index.
